# Opioid-induced constipation in patients with cancer pain in Japan (OIC-J study): a post hoc subgroup analysis of patients with gastrointestinal cancer

**DOI:** 10.1007/s10147-020-01790-y

**Published:** 2020-10-17

**Authors:** Toshiyuki Harada, Hisao Imai, Soichi Fumita, Toshio Noriyuki, Makio Gamoh, Masaharu Okamoto, Yusaku Akashi, Yoshiyuki Kizawa, Akihiro Tokoro

**Affiliations:** 1 Center for Respiratory Diseases, JCHO Hokkaido Hospital, Sapporo, Japan; 2 Division of Respiratory Medicine, Gunma Prefectural Cancer Center, Gunma, Japan; 3 Department of Respiratory Medicine, International Medical Center, Comprehensive Cancer Center, Saitama Medical University, Hidaka, Saitama Japan; 4 Department of Medical Oncology, Kindai University Nara Hospital, Nara, Japan; 5 Department of Surgery, Onomichi General Hospital, Onomichi, Hiroshima, Japan; 6 Department of Medical Oncology, Osaki Citizen Hospital, Miyagi, Japan; 7 Medical Affairs, Shionogi & Co., Ltd, Osaka, Japan; 8 Department of Palliative Medicine, Kobe University Graduate School of Medicine, Kobe, Japan; 9 Department of Psychosomatic Internal Medicine and Supportive and Palliative Care Team, National Hospital Organization Kinki-Chuo Chest Medical Center, Sakai, Osaka Japan

**Keywords:** Cancer pain, GI cancer, Observational study, Opioid-induced constipation, OIC-J

## Abstract

**Background:**

Constipation is a common side effect of opioid therapy. An observational study of opioid-induced constipation (OIC) in Japanese patients with cancer (OIC-J) included 212 patients with various tumor types. This post hoc analysis of OIC-J evaluated a subgroup of patients with gastrointestinal (GI) cancer.

**Methods:**

Patients were aged ≥ 20 years, starting strong opioid therapy, had an ECOG PS of ≤ 2, and must have had ≥ 3 bowel movements during the week before enrollment. OIC was evaluated for 2 weeks after opioid initiation using the Rome IV diagnostic criteria for colorectal disorders, as well as physician’s diagnosis, number of spontaneous bowel movements, Bowel Function Index score, and patient’s self-assessment. Relationships between baseline characteristics and OIC incidence, and the effects of OIC on quality of life (QOL) were also explored.

**Results:**

Fifty patients from OIC-J who had GI cancer [colon (50%), stomach (28%), and esophageal (22%)] were included. OIC incidence varied by which diagnostic criteria were used (46.0–62.0%) and occurred rapidly after initiating opioid therapy. The use of prophylactic laxatives reduced the overall incidence rate of OIC from 71.0% to 47.4%. No baseline characteristics, except comorbidities, were associated with OIC incidence. Change from baseline to day 15 in PAC-SYM total score was significantly greater for patients with OIC versus those without OIC (0.188 versus −0.362; *P* = 0.0011).

**Conclusions:**

This post hoc analysis suggests that OIC occurs rapidly in patients with GI cancer after initiating opioid therapy, and negatively impacts QOL. Early and effective intervention strategies may be particularly useful in this group.

**Additional Information:**

Coauthor Makio Gamoh is deceased.

**Electronic supplementary material:**

The online version of this article (10.1007/s10147-020-01790-y) contains supplementary material, which is available to authorized users.

## Introduction

Pain is a common and debilitating symptom of cancer that can cause physical and psychological suffering and has a negative impact on quality of life (QOL) [1, 2]. An estimated 55% of patients who receive anticancer treatment and 66% of patients with advanced, metastatic, or terminal disease experience pain [3]. Opioid analgesic therapy is highly effective for reducing cancer pain [4] and is recommended alone or in combination with other treatments for both the initiation and maintenance of pain relief [2]. While effective for managing cancer pain, opioid use is limited by adverse effects, which can lead the patient to discontinue the opioid medications due to a significant negative impact on QOL [4, 5].

Opioid-induced constipation (OIC), characterized by difficult‐to‐pass and hard stools, straining at defecation, and sensations of incomplete evacuation or anorectal obstruction, is a common side effect of opioid analgesic therapy [6, 7]. Criteria for OIC have been incorporated into the Rome IV diagnostic criteria for colorectal disorders [8, 9]. The Rome IV diagnostic criteria further defines OIC as new or worsening symptoms of constipation when initiating, changing, or increasing opioid analgesic therapy, and must include two or more of the following symptoms: straining, lumpy or hard stools, sensation of incomplete evacuation, sensation of anorectal blockage, use of manual maneuvers to facilitate defecation, and < 3 spontaneous bowel movements (SBMs) per week [8, 9]. Reported estimates for the prevalence of OIC vary widely (22–81%) depending on definitions and diagnostic criteria used and the type of opioid analgesic therapy administered [7]. It remains unclear, however, whether other factors such as cancer type have an impact on the reported incidence of OIC.

An observational study, Opioid-induced Constipation in Patients with Cancer Pain in Japan (OIC-J), estimated the incidence of OIC in Japanese patients with cancer after the initiation of opioid analgesic therapy [10]. The primary results of that study demonstrated that 56% of patients with cancer pain developed OIC within 2 weeks of initiating opioid analgesic therapy, although incidence varied by the type of diagnostic criteria used [10]. A secondary analysis that evaluated patients’ self-awareness of OIC found that patients recognized OIC onset after starting opioid analgesic therapy and that OIC affected both pain management and QOL [11]. The OIC-J study enrolled 50 (23.6%) patients with gastrointestinal (GI) cancer (i.e., colon, stomach, or esophageal cancers), providing an opportunity to assess whether tumors of this category have effects on the incidence of OIC [10]. This post hoc analysis reports the incidence of OIC in a subgroup of patients with GI cancer from the OIC-J study.

## Patients and Methods

### Study design

This was a post hoc subgroup analysis of data from the OIC-J study (UMIN000025864), a multicenter, prospective, observational cohort study of the incidence of OIC in Japanese patients with cancer pain who were starting strong opioid therapy. The study was approved by relevant institutional review boards and was conducted in compliance with the Declaration of Helsinki and Ethical Guidelines for Medical and Health Research Involving Human Subjects. All patients provided written informed consent. Detailed study design and methods have been previously published [10]. This post hoc subgroup analysis examined data from patients in the OIC-J study who had GI cancer.

### Key eligibility criteria

Detailed inclusion and exclusion criteria have been previously published [10]. Briefly, the OIC-J study enrolled patients aged ≥ 20 years with cancer that was expected to be stable for the duration of the study and who had an Eastern Cooperative Oncology Group performance status (ECOG PS) score ≤ 2. Eligible patients were starting strong opioid analgesic therapy and must have had ≥ 3 bowel movements during the 7 days prior to enrollment. Patients were excluded if they had any current or a history of conditions that could affect the structure and function of the GI tract or disimpaction within 7 days prior to enrollment. For this post hoc subgroup analysis, patients were included if they had GI cancer (i.e., stomach, colon, or esophageal cancers).

### Endpoints and assessments

The primary endpoint was the incidence of OIC determined by Rome IV diagnostic criteria [8]. Details of the Rome IV diagnostic criteria for OIC used in this study have been published previously [10]. Secondary endpoints included the incidence of OIC based on the attending physician’s diagnosis, occurrence of < 3 SBMs (i.e., any bowel movement with the exception of those ≤ 24 h after rescue laxatives) per week, a Bowel Function Index (BFI) score [12] of ≥ 28.8, and patient’s daily self-awareness of the presence or absence of OIC symptoms. All patients kept a handwritten paper diary for 2 weeks following initiation of opioid analgesic therapy that recorded the date and time of bowel movements; the form of stools using the Bristol Stool Scale [13]; the presence/absence of the feeling of incomplete evacuation; and the degree of straining. Patients also rated the sensation of anorectal obstruction/blockage during bowel movements on a scale from 0 (none) to 4 (very severe). Changes from baseline were measured in the Patient Assessment of Constipation Symptoms (PAC-SYM) [14, 15] and the Patient Assessment of Constipation Quality of Life (PAC-QOL) questionnaires [16]. Changes in PAC-SYM and PAC-QOL total scores from baseline to 2 weeks after starting opioid analgesic therapy were compared between patients with OIC and patients without OIC.

### Statistical analysis

The incidence of OIC was calculated as the percentage of patients with OIC during the first 2 weeks of opioid analgesic therapy. Two populations were defined for this post hoc subgroup analysis: (i) full analysis set (FAS) 1 included all enrolled patients, except those with ethical guideline violations, those with an observation period of < 4 days, and those who did not take opioids during the observation period; (ii) FAS 2 included all patients in FAS 1 with an observation period of ≥ 7 days. The incidence of OIC was assessed for FAS 1; changes from baseline in PAC-SYM and PAC-QOL total scores were assessed for FAS 2.

All statistical tests were performed on observed values, with a 2-sided significance level of 0.05 without multiplicity considerations. The Clopper–Pearson method was used to calculate 95% confidence intervals (CIs) for OIC incidence. A chi-squared test was used to test for associations between baseline characteristics and OIC onset. Change from baseline in PAC-SYM and PAC-QOL total scores were compared between patients with OIC and patients without OIC for each OIC diagnostic criterion, using Welch’s *t*-test. SAS software for Windows, Version 9.4 (SAS Institute Inc., Cary, NC), was used for data analysis.

## Results

### Patients

A total of 220 patients were enrolled in the primary OIC-J study [10], and 50 patients with GI cancer were included in this post hoc subgroup analysis. Demographic and baseline clinical characteristics are summarized in Table 1. The FAS 1 and FAS 2 populations each comprised 50 patients. Of 50 patients in FAS 1, 25 (50%) had colon cancer, 14 (28%) had stomach cancer, and 11 (22%) had esophageal cancer. The majority of patients with GI cancer were male (68%), were aged ≥ 65 years (64%), and had metastatic disease (94%).

**Table 1:** Patient demographic and baseline clinical characteristics (FAS 1 population)

Parameter	Patients with GI cancer^a^ *N* = 50	All patients *N* = 212 Tokoro et al. [10]
Sex, *n* (%)		
Male	34 (68)	145 (68)
Female	16 (32)	67 (32)
Age category, years, *n* (%)		
< 50	2 (4)	13 (6)
≥ 50, < 65	16 (32)	48 (23)
≥ 65, < 75	18 (36)	84 (40)
≥ 75	14 (28)	67 (32)
Admission status, *n* (%)		
Inpatient	18 (36)	115 (54)
Outpatient	32 (64)	97 (46)
Metastases present, *n* (%)	47 (94)	192 (91)
Anticancer medications, *n* (%)		
No	18 (36)	107 (50)
Yes	32 (64)	105 (50)
ECOG PS, *n* (%)		
0	12 (24)	51 (24)
1	31 (62)	121 (57)
2	7 (14)	40 (19)
BMs in the past week, *n* (%)		
≥ 7	17 (34)	57 (27)
7	15 (30)	63 (30)
3–6	18 (36)	92 (43)
< 3	0	0
Laxative use, *n* (%)		
Within 24 h of enrollment	5 (10)	13 (6)
Regular use before enrollment	13 (26)	56 (26)
Comorbidities, *n* (%)		
No	17 (34)	53 (25)
Yes	33 (66)	159 (75)

*BM* bowel movement, *ECOG PS* Eastern cooperative oncology group performance status, *FAS* full analysis set, *GI* gastrointestinal

^**a**^Primary tumor type: colon cancer (25 [50%] patients), stomach cancer (14 [28%] patients), and esophageal cancer (11 [22%] patients)

### Incidence and onset of OIC

The incidence of OIC, as measured using different diagnostic criteria, is summarized in Table 2. The incidence of OIC varied according to the selected criteria: 62.0% by Rome IV diagnostic criteria; 61.2% by physician’s diagnosis; 59.6% by BFI; and 46.0% by the number of SBMs.

**Table 2:** Incidence of OIC by diagnostic criteria (FAS 1 population)

Criteria	OIC incidence (*n*/*N*, %)	95% CI
Rome IV	31/50, 62.0	47.2–75.3
Physician’s diagnosis	30/49, 61.2	46.2–74.8
SBM frequency	23/50, 46.0	31.8–60.7
BFI	28/47, 59.6	44.3–73.6

*BFI* bowel function index, *CI* confidence interval, *FAS* full analysis set, *OIC* opioid-induced constipation, *SBM* spontaneous bowel movement

**Fig. 1:**
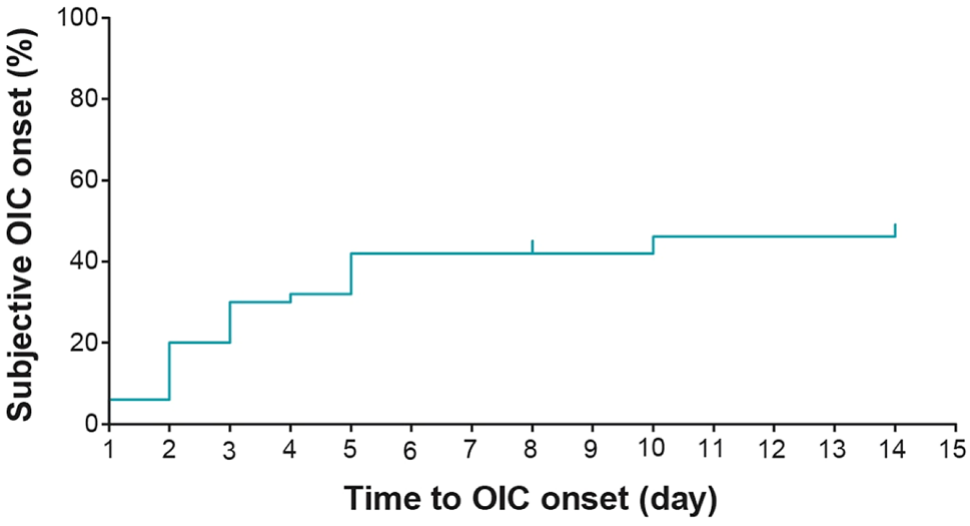
Patient awareness of OIC (FAS 1). Tick marks in the plot represent censored patients. *FAS* full analysis set, *OIC* opioid-induced constipation.

**Fig. 2:**
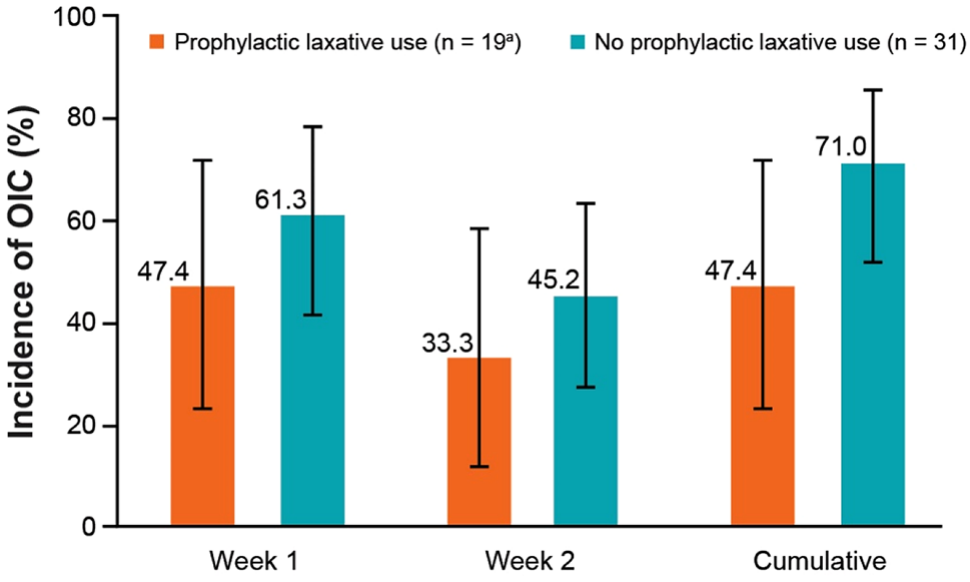
Incidence of opioid-induced constipation over the entire period of study (Rome IV diagnostic criteria) in patients with or without prophylactic laxative use (FAS 1). ^a^At the 2-week time point, *n* = 18 for patients with prophylactic laxative use. Error bars represent the 95% confidence interval. *FAS* full analysis set, *OIC* opioid-induced constipation.

The onset of OIC based on patients’ self-assessment was relatively rapid (Fig. 1), and 23 of 50 patients (46.0%) were aware of their OIC (95% confidence interval, 31.8–60.7) by 14 days after initiating opioid analgesic therapy. The use of prophylactic laxatives resulted in a reduction of the overall incidence rate of OIC from 71.0% to 47.4% (Fig. 2). The prophylactic agents for constipation included magnesium oxide (*n* = 14), sennosides (*n* = 3), naldemedine (*n* = 2), senna (*n* = 2), lubiprostone (*n* = 1), and others (*n* = 2) No patient baseline characteristics, except comorbidities, were significantly associated with OIC incidence (Table 3).

**Table 3:** Incidence of OIC (Rome IV diagnostic criteria) according to baseline characteristics in patients with GI cancer (*N* = 50; FAS 1 population)

	*n*	Incidence of OIC (%)	95% CI	χ^2^ test
Sex, *n*				
Male	34	61.8	43.6–77.8	*P* = 0.9601
Female	16	62.5	35.4–84.8	
Age category, years, *n*				
≥ 20, < 40	0	–	–	*P* = 0.4144
≥ 40, < 50	2	100.0	15.8–100.0	
≥ 50, < 65	16	62.5	35.4–84.8	
≥ 65, < 75	18	50.0	26.0–74.0	
≥ 75	14	71.4	41.9–91.6	
Admission status, *n*				
Inpatient	18	72.2	46.5–90.3	*P* = 0.2640
Outpatient	32	56.3	37.7–73.6	
Metastasis present, n				
No	3	33.3	0.8–90.6	*P* = 0.2914
Yes	47	63.8	48.5–77.3	
ECOG PS, *n*				
0	12	50.0	21.1–78.9	*P* = 0.5825
1	31	64.5	45.4–80.8	
2	7	71.4	29.0–96.3	
Anticancer medications, *n*				
No	18	66.7	41.0–86.7	*P* = 0.6101
Yes	32	59.4	40.6–76.3	
BMs in the past week, *n*				
≥ 7	17	52.9	27.8–77.0	*P* = 0.4926
7	15	60.0	32.3–83.7	
3–6	18	72.2	46.5–90.3	
< 3	0	–	–	
Regular laxative use, *n*				
No	37	67.6	50.2–82.0	*P* = 0.1712
Yes	13	46.2	19.2–74.9	
Rescue laxative use, *n*				
No	45	60.0	44.3–74.3	*P* = 0.3821
Yes	5	80.0	28.4–99.5	
Comorbidities, *n* (%)				
No	17	82.4	56.6–96.2	*P* = 0.0333
Yes	33	51.5	33.5–69.2	

*BM *bowel movement, *CI* confidence interval, *ECOG PS* Eastern cooperative oncology group performance status, *FAS* full analysis set, *GI* gastrointestinal, *OIC* opioid-induced constipation

### Relationship between PAC-SYM and PAC-QOL total score changes and OIC incidence

**Fig. 3:**
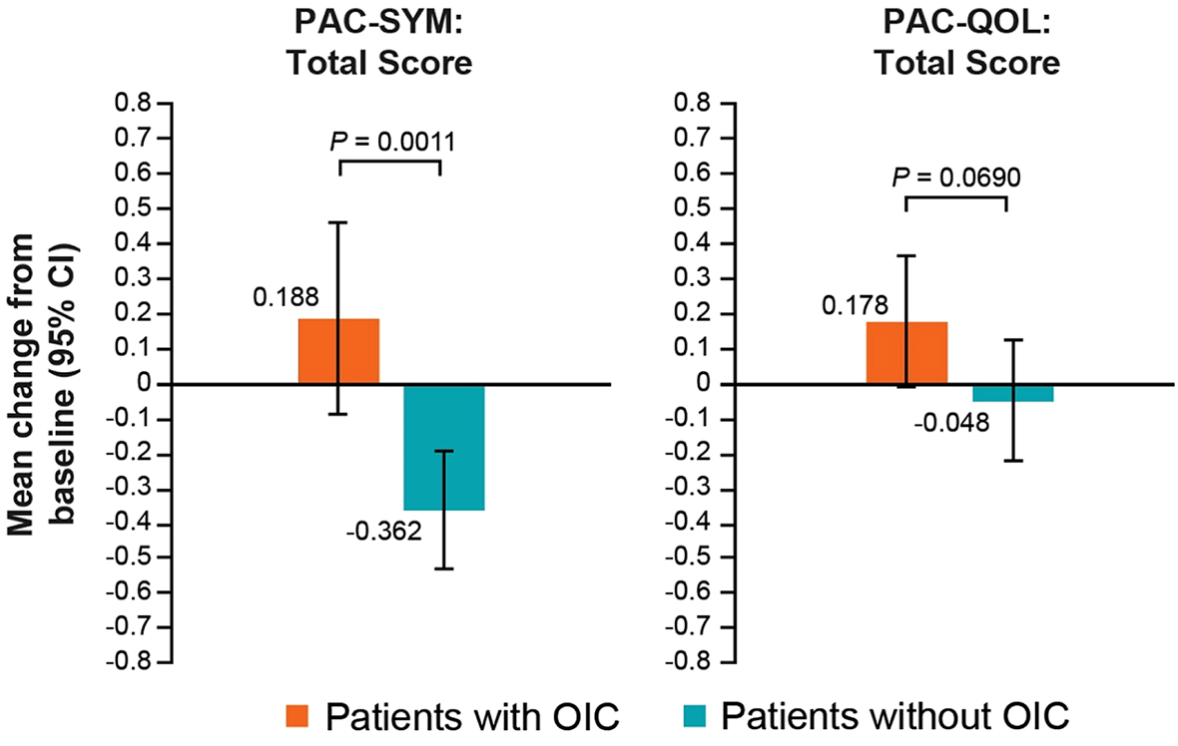
Change from baseline to day 15 in PAC-SYM and PAC-QOL total scores by Rome IV diagnostic criteria (*n* = 43; FAS 2). Error bars represent the 95% confidence interval. *CI* confidence interval, *FAS* full analysis set, *PAC-QOL* patient assessment of constipation quality of life, *PAC-SYM* patient assessment of constipation symptoms, *OIC* opioid-induced constipation.

Based on the data from PAC-SYM and PAC-QOL total scores, OIC had a negative impact on patients’ QOL (Fig. 3). By Rome IV diagnostic criteria, change from baseline to day 15 in PAC-SYM total score was significantly greater for patients with OIC versus those without OIC (0.188 versus − 0.362; *P* = 0.0011). Change from baseline to day 15 in PAC-QOL total score was numerically greater for patients with OIC versus those without OIC, although it did not reach statistical significance (0.178 versus − 0.048; *P* = 0.0690). By patients’ self-assessment, the change from baseline to day 15 in PAC-SYM and in PAC-QOL total scores were numerically greater for patients with OIC versus those without OIC, however, they did not reach statistical significance (PAC-SYM, 0.055 versus − 0.223; *P* = 0.1150; PAC-QOL, 0.178 versus −0.047; *P* = 0.0696) (Supplementary Fig. 1).

## Discussion

To our knowledge, this is the first report of OIC incidence specifically in patients with GI cancer, providing valuable data for this patient population. The results of this post hoc subgroup analysis of patients with GI cancer from a prospective, observational study demonstrates that the incidence of OIC varied depending on the diagnostic criteria used. Incidence rates for OIC were similar when assessed by Rome IV diagnostic criteria, physician’s diagnosis, and BFI (62%, 61%, and 60%, respectively). The rates of OIC incidence as measured by patients’ self-assessment and SBM were the same (both 46%).

When using the Rome IV diagnostic criteria, the incidence of OIC was numerically higher in patients with GI cancer (62%), compared with 56% among patients with different cancer types included in the primary OIC-J analysis (*n* = 212) [10]. In contrast, self-assessed incidence rates of OIC were similar between patient populations: 46% in patients with GI cancer; 48% among patients with different cancer types in the OIC-J study [11].

Patients with colon and other GI cancer commonly experience GI symptoms, including diarrhea, constipation, rectal bleeding, changes in bowel habits, abdominal cramping or pain, and an urge to have a bowel movement even with an empty bowel [17]. Therefore, a diagnosis of OIC is particularly important for this patient population who may not immediately attribute constipation to opioid analgesic use.

OIC can occur rapidly after the initiation of opioid analgesic therapy, causing discomfort and contributing to a significantly reduced QOL, highlighting the importance of early recognition and treatment of OIC. A timely diagnosis of OIC can be hindered by the level of clinical awareness surrounding the negative impact of OIC [18, 19]. In addition, patients may not be receiving adequate information from their healthcare provider (HCP) regarding the risk of OIC [19, 20]. HCP–patient communication regarding the risk of OIC may be particularly useful for patients with GI cancer, as they may have concomitant cancer-related GI symptoms. The use of prophylactic laxatives was associated with a decrease in the incidence of OIC in this subgroup analysis and in the primary patient population of the OIC-J study [10].

A limitation of this study is the exclusion of patients who had any current or cured conditions that could affect GI tract structure or function, resulting in a study population that may be different from the general population of patients with GI cancer. Other limitations include the post hoc study design and the relatively small number of patients included.

In conclusion, results from this post hoc, subgroup analysis demonstrate that approximately half of patients with GI cancer who initiated opioid analgesic therapy developed OIC, with the exact incidence dependent on which diagnostic criteria were used. In patients with GI cancer, OIC occurred rapidly after the initiation of opioid analgesic therapy and had a negative impact on patient QOL. Early and effective intervention strategies such as prophylactic laxatives may be particularly useful in this patient population. Early intervention strategies for patients with GI cancer include methods to prevent constipation and to carefully observe and monitor a patient’s condition. This may be achieved in part with the use of prophylactic laxatives. Treatment guidelines from the National Comprehensive Cancer Network and the European Society for Medical Oncology recommend the use of laxatives and self-care strategies (eg, exercise therapy, maintaining adequate fluid intake and dietary fiber) for the prevention and management of constipation [21, 22].

## Electronic supplementary material

Below is the link to the electronic supplementary material.

Supplementary file1 (JPG 1480 kb)
