# Supplementary figures and images for: Opioid-induced constipation in patients with cancer pain in Japan (OIC-J study): a post hoc subgroup analysis of patients with gastrointestinal cancer
Source: Int J Clin Oncol. 2020 Oct 17;26(1):104–10. doi: 10.1007/s10147-020-01790-y (PMC7788034; doi:10.1007/s10147-020-01790-y)

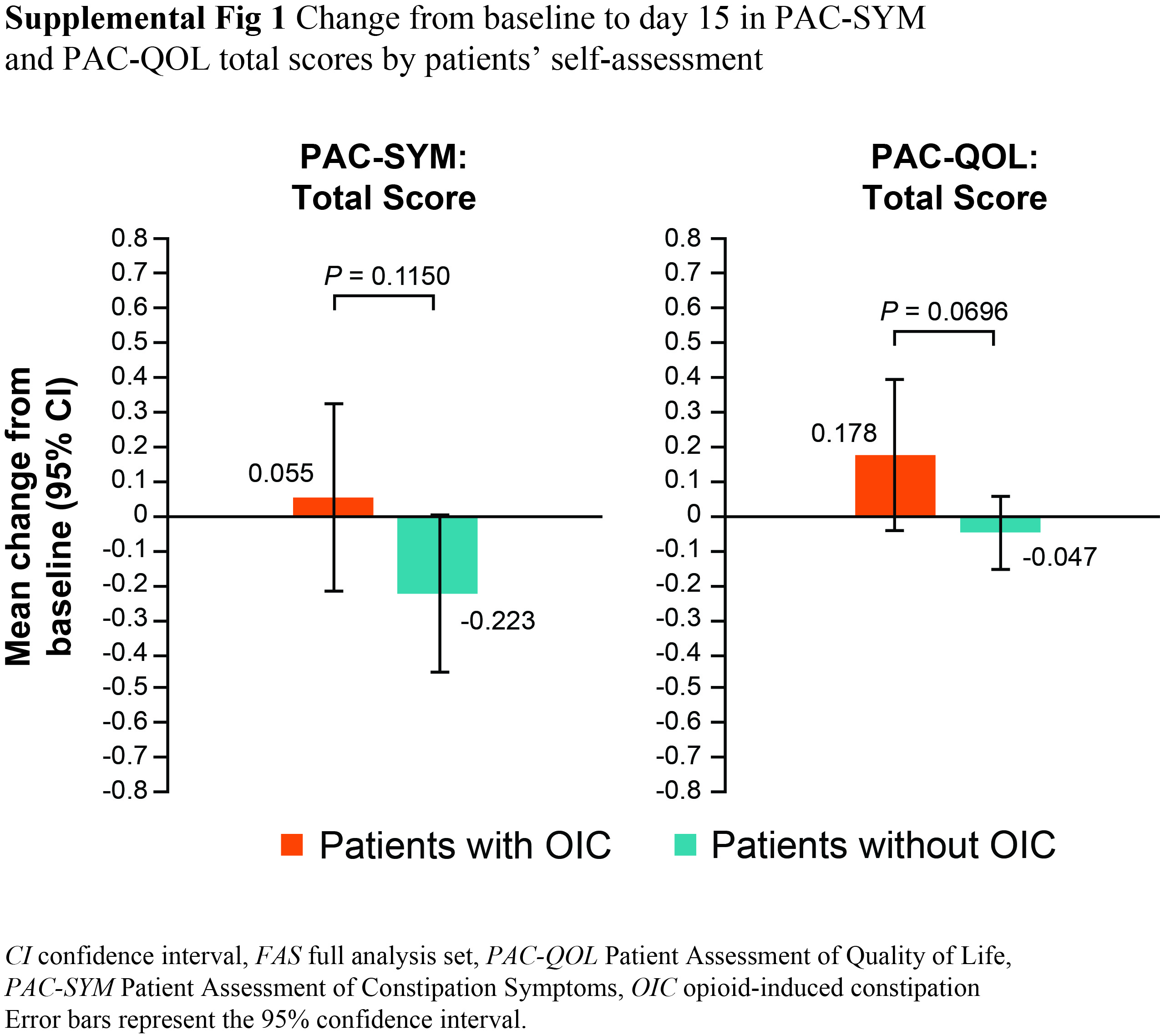

Supplement: Supplementary file 1 — Supplementary file1 (JPG 1480 kb) [file 10147_2020_1790_MOESM1_ESM.jpg]
